# Hormonal contraception, sexual behaviour and HIV prevalence among women in Cameroon

**DOI:** 10.1186/1472-6874-8-19

**Published:** 2008-10-30

**Authors:** Eugene J Kongnyuy, Varda Soskolne, Bella Adler

**Affiliations:** 1 Child and Reproductive Health Group, Liverpool School of Tropical Medicine, UK; 2 School of Social Work, Bar-Ilan 2 University, Ramat-Gan, Israel; 3 Braun School of Public Health, Hadassah – Hebrew University, Jerusalem, Israel

## Abstract

**Background:**

Data on the effect of contraceptive methods, other than the condom, on HIV acquisition is not clear. The aim of this study was to describe hormonal contraceptive use, sexual behaviour and HIV prevalence among women in Cameroon in order to provide baseline information for future analytical studies.

**Methods:**

This is a cross-sectional descriptive study based a nationally representative sample of 4486 sexually active women aged 15–49 years who participated in the 2004 Cameroon Demographic and Health Survey.

**Results:**

The overall HIV prevalence was 7.4% (332/4486). The HIV prevalence was higher in the 25–35 year age group (10.03%), urban residents (9.39%), and formerly married (18.48%), compared to their compatriots. The prevalence was lower in women with five or more living child (3.67%), women in the low wealth index category (3.79%) and women who had no formal education (3.37%). The HIV prevalence was higher among women who had two or more partners in the last 12 months (10.26%) and women who reported to have had four or more partners in their lifetime (12.40%). The prevalence of HIV was higher among current hormonal contraceptive users (6.63%) compared to the current non-users (3.06%), among ever users of hormonal contraception (13.27%) compared to the never users (7.11%).

**Conclusion:**

We conclude that the prevalence of HIV among sexually active women in Cameroon varies according to sociodemographic characteristics, sexual behaviour and hormonal contraceptive use. Our findings underscore the need to counsel women using hormonal contraception to be aware that hormonal methods do not protect against HIV infection. Given the biologic plausibility of the link between hormonal contraception and HIV infection, future research should focus on carefully designed prospective studies to establish the temporal relationship and estimate the incidence of HIV infection among women using and not using hormonal contraceptive methods.

## Background

Acquired Immunodeficiency Syndrome (AIDS) has claimed more than 25 million lives since its discovery in 1981 [1]. Today, about 33.2 million people live with Human Immunodeficiency Virus (HIV) – the virus that causes AIDS – infections. Sub-Saharan Africa has just over 10% of the world's population, but 64% of people living with HIV, and 77% of all women with HIV worldwide live in this region [1,2]. Cameroon is one of the hardest-hit countries in Sub-Saharan Africa with an overall adult prevalence of 5.5% [3].

The main mode of HIV transmission in Sub-Saharan Africa is via heterosexual sex. The major factors associated with HIV transmission include the presence of other sexually transmitted infections (STIs), and sexual risk behaviors, such as multiple sexual partnerships, commercial sex, and the non-use of condoms. These behaviors are affected by the individual lack of knowledge about HIV, ignorance of individual HIV status, beliefs about HIV/AIDS, cultural factors such as female circumcision, widow inheritance, as well as by societal structural factors such as poverty, migration, or gender inequalities [4-6].

Less is known about the effect of various contraceptive methods on the risk of HIV acquisition. Recent evidence suggests that some contraceptive methods could be protective against HIV infection while others could increase the risk of HIV transmission [7-12]. However, apart from the condom which has demonstrated substantial protection against HIV infection, the effect of the other methods on HIV transmission remains largely unknown [8,9]. Theoretically, hormonal contraceptives could predispose women to HIV infection by increasing vaginal HIV receptors [10], causing thinning of the vaginal epithelium [11], or increasing the risk of chlamydial infection [2].

The aim of the current study was to describe hormonal contraceptive use, sexual behaviour and HIV prevalence among women in Cameroon in order to provide baseline information for future analytical studies.

## Methods

### Study Design

This cross-sectional descriptive study is based on data from the 2004 DHS. The survey was approved by the Ethics Committee of the ORC Macro at Calverton, Maryland, USA and by the National Ethics Committee in the Ministry of Health in Cameroon. All study participants gave informed consent before participation and all information was collected confidentially.

### Sampling technique

Methods used in the Cameroon DHS have been published elsewhere [3]. Briefly, the survey used a two-stage cluster sampling technique. The country was stratified into 12 domains (10 provinces and 2 major cities). Each domain is made up of enumeration areas (EAs) established by a General Census of Population and Housing in 2003. The sample frame was a list of all EAs (clusters). Within each domain, a two-stage sample was selected. The first stage involved selecting 466 clusters (EAs – primary sampling units) with a probability proportional to the number of households in the cluster. The second stage involved the systematic sampling of households from the selected clusters. All women aged 15 to 49 years in the selected households were interviewed.

### Study population

Of the total 10656 women who participated in the survey, 4,538 sexually active women were also tested for HIV and all are included in the current study.

### Data Collection

After obtaining an informed consent from the women, data were collected by face-to-face interviews at home using a structured questionnaire. Blood sample for HIV testing was drawn after the interview. For the current study we extracted information on sociodemographic characteristics, sexual behavior, contraceptive use and HIV status.

### Variables

We used six characteristics to define socio-demographic characteristics: (a) age (15–24, 25–34 or 35–49 years), (b) place of residence (rural or urban), (c) marital status (never married, currently married, divorced/widowed), (d) number of children (0, 1–2, 3–4 or ≥ 5), (e) education (no schooling, primary and secondary/higher) and (f) wealth index (low, middle or high). Wealth index was defined based on the availability of some household amenities. A score was attributed to each household amenity and the total score constituted the wealth index score [13]. We divided this score into three equal classes of wealth based percentiles, that is, less than 33.33th percentile (low, i.e. poorest), 33.33 to 66.66th percentile (medium, i.e. moderately rich), and more than 66.66th percentile (high, i.e. richest).

Sexual behavior was defined by three variables: (a) age at sexual debut (≤ 14, 15–17 or ≥ 18 years; (b) number of sexual partners in the last 12 months (0, 1 and ≥ 2 partners); and (c) life number of partners (1, 2–3 and ≥ 4 partners).

Two variables were used to define hormonal contraceptive use: (a) current use (current user or current non-user) and (b) ever use (ever user or never user). Current user referred to a woman was using the contraceptive at the time of interview, and ever user referred to a woman who had taken the contraceptive at any one point in time since sexual debut including the time when she was interviewed. Hormonal contraception was defined as the use of oral contraceptive pills, injectable contraceptives or implants.

HIV status was determined by the positive or negative results of the HIV test, based on the presence of specific anti-HIV antibodies in the blood sample. Direct ELISA (Genscreen Plus version, BioRad Laboratories) was used to screen for both HIV1 and HIV2. Positive results were confirmed by competitive ELISA (Wellcozyme HIV-1 recombinant, ABBOTT) specific for HIV-1 and a rapid test (Determine, ABBOTT) specific for HIV-2. All positive and doubtful results were finally confirmed with the Western Blot test.

### Statistical analyses

All cases in the DHS data were given weights to adjust for differences in probability of selection of subjects and to adjust for the non-response in order to produce a proper representation of the whole country [3]. The weight is determined such that it is inversely proportional to the response rate as well as the probability of selection. Therefore, the use of weights corrects for the differential response rates and the unequal probability used to select subjects in the sample. We used individual weights data analysis in this study.

Data were entered and analyzed with the Statistical Package for Social Sciences (SPSS) software programme (version 15.0, Chicago, IL, USA). The prevalence of HIV was determined for different categories of socio-demographic variables, sexual behaviour and hormonal contraceptive use. The results were expressed in terms of proportions or percentages with their corresponding 95% Confidence Intervals (CI).

## Results

Of the 4486 women who participated in the study, 332 (7.4%) were HIV positive, 136 (3.0%) were current hormonal contraceptive users and 211 (4.7%) were ever users of hormonal contraception.

### Socio-demographic characteristics

The prevalence of HIV was higher in the 25–35 year age group (10.03%, 95% Confidence Interval [CI] 8.59 to 11.63), compared to the 15–24 year age group (5.91%, 95%CI 4.88 to 7.10) and 35–49 year age group (6.38%, 95%CI 5.11 to 7.84). HIV prevalence was higher among women who lived in the urban area (9.39%, 95%CI 8.27 to 10.60%), compared to women who lived in the rural area (5.10%, 95%CI 4.22 to 6.11). HIV prevalence was 18.48% (95%CI 15.03 to 22.34) among formerly married (widowed and divorced) and this figure was 3-fold higher than the prevalence among never married and currently married women. The HIV prevalence was lower in women with five or more living child (3.67%, 95%CI 2.62 to 4.99), women in the low wealth index category (3.79%, 95%CI 2.92 to 4.83) and women who had no formal education (3.37%, 95%CI 2.41 to 4.59), compared to women with fewer children, middle and higher wealth index and women who had been to school respectively. Table 1 presents the distribution of socio-demographic characteristics by HIV prevalence among women in Cameroon.

**Table 1:** The distribution of socio-demographics, sexual behaviour and hormonal contraceptive use by HIV status among Cameroonian women (N = 4486)

	**HIV Positive**		
**Variable**	***Number***	***Percent***	***95% Confidence interval***

***1. Socio-demographic***			
*Age (years)*			
15–24	103/1742	5.91	4.88–7.10
25–34	151/1505	10.03	8.59–11.63
35–49	79/1239	6.38	5.11–7.84
*Place of residence*			
Rural	106/2078	5.10	4.22–6.11
Urban	226/2407	9.39	8.27–10.60
*Marital status*			
Never married	37/566	6.54	4.71–8.81
Currently married	215/3487	6.17	5.40–7.00
Formerly married	80/433	18.48	15.03–22.34
*No. of living children*			
0	84/952	8.82	7.14–10.75
1–2	146/1575	9.27	7.91–10.78
3–4	69/978	7.06	5.57–8.79
≥ 5	36/982	3.67	2.62–4.99
*Wealth index*			
Low	59/1557	3.79	2.92–4.83
Middle	142/1520	9.34	7.95–10.88
High	132/1409	9.37	7.93–10.97
*Educational level*			
No schooling	36/1067	3.37	2.41–4.59
Primary	146/1767	8.26	7.05–9.62
Secondary or higher	155/1811	8.56	7.34–9.91
***2. Sexual behaviours***			
*No. of partners in the last 12 months*			
0	52/594	8.75	6.68–11.23
1	248/3578	6.93	6.13–7.80
≥ 2	32/312	10.26	7.24–14.00
*Lifetime No. of partners*			
1	41/1616	2.54	1.85–3.39
2–3	137/1629	8.41	7.13–9.83
≥ 4	153/1234	12.40	10.65–14.33
***3. Hormonal contraception***			
*Current hormonal contraceptive use*			
Current non-users	127/4154	3.06	2.57–3.61
Current users	9/136	6.62	3.28–11.79
*Ever use of hormonal contraception*			
Never users	304/4275	7.11	6.37–7.91
Ever users	28/211	13.27	9.18–18.36

### Sexual behavior

The HIV prevalence was higher among women who had two or more partners in the last 12 months (10.26%, 95%CI 7.24 to 14.00) compared to their compatriots with zero or one partner (Table 1). Similarly, the HIV prevalence was higher in women who had had four or more partners in their lifetime (12.40%, 95%CI 10.65 to 14.33) compared to women with fewer number of lifetime sexual partners. The HIV prevalence did not differ by age at sexual debut among women of reproductive age.

### Hormonal contraceptive use

The prevalence of HIV was higher among current hormonal contraceptive users (6.63%, 95%CI 3.28 to 11.79) compared to the current non-users (3.06%, 9%CI 2.57 to 3.61). Similarly, the prevalence of HIV was higher among the ever users of hormonal contraception (13.27%, 95%CI 9.18 to 18.36%) compared to the never users (7.11%, 95%CI 6.37% to 7.91).

## Discussion

In this study we sought to describe the distribution of socio-demographic characteristics, sexual behaviour and hormonal contractive use by HIV status among women of reproductive age in Cameroon using data from a population-based nationally representative survey. The use of hormonal contraception among the women was low (3.0%) and HIV prevalence rate (7.4%) was higher than previously reported [3], due in part that the current study included only sexually active women.

The low level of hormonal contraceptive use among Cameroonian women of reproductive age was expected, based on the known high fertility rate in Cameroon: 176 births per 1000 women aged 15 to 44 years [3]. Similar low rates of contraceptive use have been reported in other sub-Saharan African countries, ranging from as low as 2.5% in Chad to 25.7% in Malawi [14,15]. Therefore there is need for more easily accessible family planning services and education of women and men about family planning in Cameroon.

In the current study HIV prevalence was found to higher among formerly married women, women living in urban areas and 25–34 year age group. These findings are similar to findings of previous studies in other African countries [16,17]. We found the HIV prevalence to be higher among educated women which is similar to the findings of one study [18], but differed from the findings of another study [17]. The findings of the current study affirm the known significant positive correlation between the number of wealth and sexual partners on the one hand and the risk of HIV infection of the other hand [19,20].

The current study found the HIV prevalence to be higher among current and ever users of hormonal contraceptives, compared to their compatriots. One cross-sectional study found a significantly higher HIV prevalence among women on depo-medroxyprogesterone acetate (DMPA) and oral contraceptives in four countries in Africa [21]. However evidence from prospective cohort studies suggests that DMPA and oral contraceptives are not associated with increased risk of HIV infection [9,22]. Current evidence suggests that there is no overall risk of HIV-1 acquisition among women taking hormonal contraception [23]. To date no randomized controlled trials have been conducted because of arguments that randomizing women who desire hormonal contraceptive could be unethical [23]. Therefore the potential for hormonal contraception to increase HIV acquisition remains unclear.

The methodology of the current study poses some limitations. Self-reported use of hormonal contraception and sexual behavior may be biased in ways that affect the prevalence of HIV among different groups. Furthermore, all hormonal contraceptives were put into one category although HIV prevalence may differ from one type of hormonal contraceptive to another.

Despite these limitations, this study has several strengths. Firstly, the study population is a national representative sample which allows generalization of the findings to all Cameroonian women of reproductive age. Lastly, many of the previous studies relied on rapid tests for HIV determination. Our study however used two ELISA tests followed by confirmation with Western Blot.

## Conclusion

In conclusion, we found that the prevalence of HIV in women of reproductive age was higher among urban residents, formerly married, wealthier women, educated women, women with 2 or more partners and current users of hormonal contraception, compared to their compatriots. The findings, however, have several important implications for practice and further research: (a) women in Cameroon using hormonal contraception should be well informed that the hormonal methods do not protect against HIV infection; (b) prevention efforts should be focused on skills training in proper use of condoms, consolidating consistent condom use and preventing relapse, not only raising awareness; (c) future research should focus on carefully designed prospective studies to establish the temporal relationship and estimate the incidence of HIV infection among women using different contraceptive methods.

## Competing interests

The authors declare that they have no competing interests.

## Authors' contributions

EJK conceived the study, extracted the data, did the analyses and interpretation, and wrote the first draft of the manuscript. VS participated in the interpretation and critically revised the manuscript for important intellectual content. BA helped in the analysis of data and interpretation. All authors read and approved the final manuscript.

## Pre-publication history

The pre-publication history for this paper can be accessed here:


